# Natural drugs targeting inflammation pathways can be used to treat atherosclerosis

**DOI:** 10.3389/fphar.2022.998944

**Published:** 2022-11-01

**Authors:** Xiayinan Song, Xiaoming Wang, Danyang Wang, Zhenzhen Zheng, Jie Li, Yunlun Li

**Affiliations:** ^1^ Innovative Institute of Chinese Medicine and Pharmacy, Shandong University of Traditional Chinese Medicine Jinan, Jinan, China; ^2^ Experimental Center, Shandong University of Traditional Chinese Medicine, Jinan, China; ^3^ Affiliated Zhongshan Hospital of Dalian University, Dalian, China; ^4^ Department of Cardiology, Affiliated Hospital of Shandong University of Traditional Chinese Medicine, Jinan, China

**Keywords:** atherosclerosis, inflammation, natural drugs, interleukin, TCM

## Abstract

Atherosclerosis (AS) is the chronic gradual degradation of arteries in combination with inflammation. Currently, the main research focus has been on interactions between inflammatory cells, inflammatory mediators, and immune mechanisms, while some studies have reported natural drugs were exerting a critical role against AS, whereas the usage of natural drugs was always limited by various factors such as poor penetration across biological barriers, low bioavailability, and unclear mechanisms. Herein, we reviewed the potential targets for inflammation against AS, discussed the underlying mechanisms of natural drugs for AS, particularly highlighted the dilemma of current research, and finally, offered perspectives in this field.

## Introduction

Atherosclerosis (AS) is a major medical and social issue, with significant clinical morbidity and mortality manifestations (Libby, 2021). AS is a multifactorial disease characterized by degenerative changes in the aorta wall, followed by lumen occlusion and restricted blood supply to organs and tissues. Subclinical (asymptomatic) AS has the most extensive pathology. It is accepted that many young individuals have atherosclerotic lesions that have developed for decades until the clinical manifestation. In middle age, individuals usually do not have AS clinical manifestations, and in fact, the incidence of atherosclerotic lesions accounts for almost 100% (Insull, 2009). However, current treatment approaches using lipid-lowering statins prevent only 65% of all cardiovascular events (Ridker et al., 2009).

**FIGURE 1:**
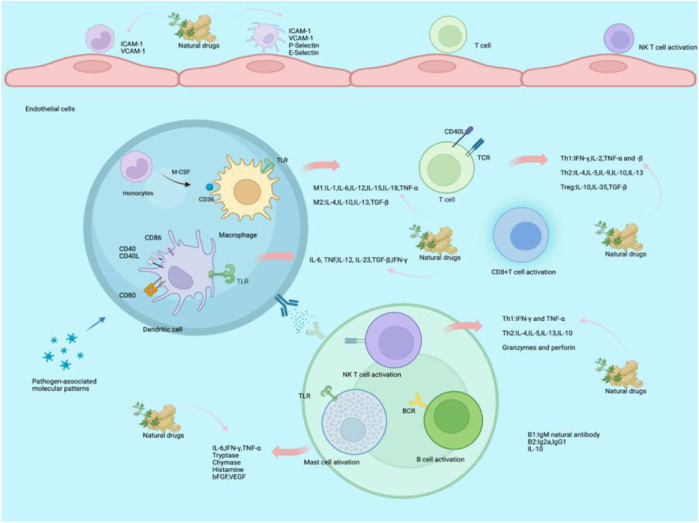
Mechanism of natural drugs on atherosclerotic inflammatory factors. At the early stage of AS, monocytes are tethered through the interaction between monocyte P-selectin glycoprotein-1 and endothelial P-selectin. With regard to adhesion and exudation, monocytes express very late antigen 4 (VLA4) and lymphocyte function-associated antigen-1 (LFA-1) to bind endothelial cell ligands, including VCAM-1 and ICAM-1. The monocytes differentiate into macrophages by M-CSF mediators. After activation, monocytes differentiate into two main phenotypes of macrophages: inflammatory M1 and regulatory M2 macrophages. M1 macrophages produce inflammation by secreting pro-inflammatory cytokines after intake of modified LDL and present antigens to T cells through macrophages using pattern-recognition receptors (PRRs), resulting in releasing pro-inflammatory cytokines to activate T cells, including IL-1, IL-6, IL-12, IL-15, IL-18, MIF, and TNF-α. M2 macrophages have anti-inflammatory functions to address plaque inflammation through efferocytosis and the release of Th2 cytokines such as IL-4, IL-10, and IL-13.

Several theories have been expounded on the cause of AS. Early studies suggested that AS was mostly associated with lipid content, growth factors, and smooth muscle proliferation (Davies, 1994). However, in the past decades, scientists have become increasingly aware of inflammation factors in AS and its associated complications. Critically, inflammation is a trigger, which leads to plaque formation and development, as shown in Figure 1.

In recent years, the multi-target effects of natural drugs have attracted the attention of globe researchers. Studies have found that natural drugs could not only directly participate in the inflammatory process of AS (regulating monocytes, macrophages, and lymphocytes) but also indirectly regulate pro-inflammatory factors, including tumor necrosis factor-α (TNF-α), angiotensin (Ang)-II, interferon-γ (IFN-γ), interleukin-1 (IL-1), IL-6, vascular cellular adhesion molecule-1 (VCAM-1), intracellular adhesion molecule-1 (ICAM-1), and C-reactive protein (CRP). However, the use of natural drugs was always limited by several factors. For instance, repeated administration of natural drugs at high doses is always required and, as a result, may induce a series of side effects. Furthermore, most natural drugs are always characterized by low stability, poor penetration into the diseased site, and limited ability to cross cell membranes.

In this review, we summarized and discussed the regulatory and reparative effects of natural drugs on inflammation processes during AS occurrence and development. We discussed their merits for AS and their underlying mechanisms toward inflammation in AS. Last but not the least, in the course of this study, we found various problems in the research process of natural drugs and also put forward better opinions providing a clearer opinion for future research.

## Alkaloids

### Tetramethylpyrazine

Tetramethylpyrazine (TMP) (2,3,5,6-tetramethylpyrazine) is an active ingredient of traditional Chinese medicine (TCM): *Ligusticum chuanxiong* has been used to treat AS for decades. It has been reported that TMP can dilate blood vessels, increase blood flow in coronary arteries and other organs, and accelerate microcirculation, which can effectively prevent thrombosis, so it is widely used in various ischemic diseases, including ischemia–reperfusion. In addition, in view of the protective effect of TMP on vascular endothelial cells (VECs), it can be used as a potential developing drug for the treatment of AS (Zhang et al., 2017). The effects of TMP on critical AS components have been intensively investigated. Sun et al. (2018) reported that TMP inhibited AS in ferroportin-1 Tek-Cre mice fed a high-cholesterol diet (HCD) by inhibiting hepcidin, nitric oxide (NO), endothelin-1 (ET-1), reactive oxygen species (ROS), malondialdehyde (MDA), superoxide dismutase (SOD), IL-1, IL-6, and TNF-α. Furthermore, Wu et al. (2012) demonstrated that TMP could improve vascular endothelial dysfunction caused by AS and achieve immunomodulatory effects on endothelial cells by inhibiting ICAM-1 and heat shock protein 60 (HSP60). These results suggested that TMP protects endothelial cells by reducing inflammatory factors and suppressing immune responses, thereby preventing AS.

### Berberine

Berberine (BBR) is an active ingredient extracted from *Coptis*, a TCM. Recent research reported that BBR exerted good therapeutic effects in AS. Wan et al. (2018) showed that BBR reduced adipsin, lipid, IL-6, and TNF-α serum levels in mice, decreased adipsin, phosphor-p38 (p-p38) mitogen-activated protein kinase (MAPK), and p-c-Jun N-terminal kinase (JNK) protein expression in mice aorta, and reduced adipin distribution in atherosclerotic lesions in apolipoprotein-E knockout Apolipoprotein-E knockout [ApoE (−/−)] mice. BBR reduced blood lipid levels, aortic ROS production, and serum levels of MDA, oxidized low-density lipoprotein (ox-LDL), and IL-6 in ApoE^−/−^ mice, thereby reducing carotid atherosclerotic lesions in mice (Tan et al., 2020). BBR regulated lipid homeostasis and inhibited macrophage foam cell formation by inhibiting activator protein-1 (AP-1) activity and activation of the nuclear factor erythroid 2-related factor 2 (NRF2)/heme oxygenase-1 (HO-1) pathway (Yang et al., 2020). Elevated extracellular matrix metalloproteinase inducer (EMMPRIN) and matrix metalloproteinase-9 (MMP-9) levels in ox-LDL-induced macrophages caused vulnerable plaques *via* extracellular matrix degradation, while BBR inhibited ox-LDL-induced AMP-activated protein kinase (AMPK)-α and MAPK signaling in macrophages by increasing miR150-5p levels; therefore, P2X7 receptor-mediated (P2X7R) EMMPRIN and MMP-9 expression was inhibited (Lu et al., 2021). In the experiment of the innovative technology BBR-mediated sonodynamic therapy (BBR-SDT) on human leukemia monocytic cell line (THP-1) macrophages, macrophage autophagy was increased after treatment and foam cell autophagy resistance was blocked, inducing cholesterol efflux (Kou et al., 2017). BBR attenuated ox-LDL-induced monocyte adhesion to HUVECs by inhibiting VCAM-1 and ICAM-1 expression, thereby indicating that BBR provided protective roles in early AS stages (Huang et al., 2013). A BBR-embedded nanosystem reduced TNF-α, IL-6, IL-1β, IFN-γ, monocyte chemotactic protein (MCP), and macrophage inflammatory factor (MIP) plasma levels (Ma et al., 2020).

### Piperlongumine

*Piperlongumine* (PL) is a natural small-molecule alkaloid isolated from *Piper longum*. PL inhibited the migration and proliferation of vascular cells produced by atherosclerotic lesions by inhibiting platelet-derived growth factor-BB (PDGF-BB) and NF-kB and inhibited the occurrence and development of plaques in AS (Son et al., 2012).

## Glycosides

### Panax notoginseng saponins

*Panax notoginseng* is the dry root and rhizome from *Panax notoginseng* [*panax notoginseng* (*Burk*.) *F.H. Chen*]. According to reports, the different anatomical sections of *Panax notoginseng* (roots, stems, leaves, flower buds, and seeds) contain many different saponins, mainly ginsenosides (Rb1\Re\Rg1\Rg2\Rh1), notoginseng saponins (R1–R6), and aescin (VII) (Zhou et al., 2017). In recent years, tablets, capsules, and injections with total *Panax notoginseng saponins*’ (PNS) as the main components have been formulated. With a market value of billions of Yuan, notoginseng total glycosides tablets, Xuesaitong injections, and Xueshuantong injections have been successfully used to treat cardiovascular diseases (Zhou et al., 2017). Yang et al. (2022) reported that after PNS administration for 8 weeks, nuclear factor-κB (NF-κB) p65, IL-6, IL-1β, TNF-α, and calpain1 protein expression levels in aortic root tissues in ApoE^−/−^ mice were inhibited *via* NF-κB signaling (Yang et al., 2022). Fan et al. (2012) reported that PNS reduced the size of plaques on the aorta of AS rats by upregulating lipid metabolism by upregulating LXRa; at the same time, the expressions of NF-kB, IL-6, and MCP-1 in blood vessels were also inhibited. Yuan et al. (2011) reported that intraperitoneal injection of PNS could improve AS induced by zymosan A. PNS reduced aortic platelet plaque in model rats and inhibited the formation of foam cells, most integrin families, focal adhesion kinase (FAK) threonine 397 phosphorylation, and NF-kB transcription. Zhang et al. (2008a) discovered that the expression of inflammation-related genes (ICAM-1, VCAM-1, CCL2, CCR2, MMP-2, MMP-9, IL-18, and IL-1β) and vasoactive factor genes [ET-1, ET2, ET3, and coagulation factor III (TF)] was decreased after a 9-week treatment with PNS. Lin et al. reported that PNS inhibited oxidative stress and attenuated the expression of inflammatory cytokines in AS through the advanced glycation end product (RAGE)/MAPK signaling pathway. The authors believed that the reduction of atherosclerotic plaque by PNS mainly depended on reducing inflammation (Dou et al., 2012). *In vivo* studies showed that PNS inhibited monocyte adhesion on an activated endothelium and the expression of TNF-α-induced endothelial adhesion molecules, such as ICAM-1 and VCAM-1, in a dose-dependent manner. Notably, IL-6 and TNF-α serum levels in all PNS treatment mouse groups were below detection limits and the serum lipid levels were also suppressed (Wan et al., 2009).

### Notoginsenoside R1

*Notoginseng saponin R1* (NGR1) is a monomeric component isolated from PNS. Geniposide combined with NGR1 (GN combination) reduced inflammation and apoptosis in AS *via* the AMPK mechanistic target of the rapamycin (mTOR)/Nrf2 signaling pathway. NOD-, LRR- and pyrin domain containing 3 (NLRP3), caspase-1, IL-1β, and IL-18 expression levels in the aortic tissue of mice were inhibited, while *in vitro* studies showed that the GN combination inhibited hydrogen peroxide (H_2_O_2_)-induced inflammatory responses and HUVEC apoptosis (Liu et al., 2021). Jia *et al.* showed that NGR1 markedly reduced inflammatory cytokine levels, including IL-2, IL-6, TNF-α, and IFN-γ in ApoE^−/−^ mice (Jia et al., 2014). Zhao et al. (2020a) found that NGR1 mitigated ox-LDL-induced apoptosis, oxidative stress, and inflammatory factor release in HUVECs by modulating the X-inactive specific transcript (XIST)/miR-221-3p/TNF-receptor-associated factor 6 (TRAF6) axis. Zhu et al. (2020) indicated that HUVECs pretreated with 30 µM of NGR1 can reduce the levels of inflammatory cytokines IL-6 and IL-1β induced by ox-LDL. The mechanism could be NGR1 upregulated miR-221-3p expression to inactivate the toll-like receptor 4 (TLR4)/NF-kB pathway. Another study also showed that NGR1 pretreatment at 30 µM enhanced NGR1-induced migration inhibition and MCP-1 and ICAM-1 downregulation by downregulating miR-132, thereby preventing ox-LDL-induced atherosclerotic responses (Fu et al., 2018).

### Icariin

Icariin (ICA) is a flavonoid isolated from the TCM herb *Epimedium brevicornum Maxim*. ICA commonly exerts multiple effects such as sex hormone regulation and relieving AS and antioxidant activity. Hu et al. (2016) showed that ICA suppressed levels of IL-6, TNF-α, mRNA, and p-p38 MAPK by reducing oxidative stress and inflammation associated with p38 MAPK signaling in rats fed with HCD. Yang et al. (2015) reported that 4 or 20 µM ICA treatment could upregulate the expression of scavenger receptor class B type I (SR-BI) protein and downregulate the expression of CD36 in a dose-dependent manner, respectively, thereby inhibiting the formation of foam cells. ICA also reduced CX3CR1 and CX3CL1 protein levels in artery walls (Wang et al., 2016a). Similarly, 10, 20, and 40 μmol/L ICA limited oxidative damage and monocyte adhesion to HUVECs and reduced the secretion and expression of ICAM-1, VCAM-1, and E-selectin (E-sel) (Hu et al., 2015).

### Paeono

The active components of *Cortex Moutan* mainly include phenols, monoterpenes, their glycosides, triterpenes, flavonoids, tannins, steroids, etc. Among them, the content of paeonol is relatively high, which is the content determination index of *Cortex Moutan*. Chen et al. (2014) reported that in their previous research paeono (Pae) was used alone on vascular smooth muscle cells (VSMC) or VECs and the preventive effect of Pae on AS was found. With the deepening of the experiment, it was found that the prevention and treatment mechanism of Pae on AS was due to the inhibition of vascular endothelial growth factor (VEGF) and PDGF-B secretion in VECs and the inhibition of Ras/Raf/extracellular signal-regulated kinase 1/2 (ERK1/2) signaling pathway in VSMCs. Yuan et al. (2016) reported that Pae inhibited monocyte adhesion to ox-LDL-damaged VECs and blocked the activation of phosphatidylinositol 3-kinase (PI3K)/protein kinase B (Akt)/NF-κB signaling pathway by promoting miR-126 expression. In the meantime, VCAM-1 expression was inhibited. The authors suggested that miR-126 could earn an irreplaceable position in the process of Pae targeting vascular inflammation in the treatment of AS.

### Diosgenin

Diosgenin is a *phytosteroid* saponin that comes from a wide range of sources, such as *Dioscorea*, fenugreek, Smilax, woodsy, and holly, in particular fenugreek and *Dioscorea* seeds. Diosgenin is also one of the main components in the eight steroidal saponins in Di’ao Xinxuekang preparations. Diosgenin inhibited inhibitor of nuclear factor kappa-B kinase beta (IKKβ)-phosphorylation and downregulated the expression of TNF-α, IL-6, MCP-1, and inducible nitric oxide synthase (iNOS) and protected endothelial function against inflammatory damage (Liu et al., 2012; Chen et al., 2016). Diosgenin also significantly inhibited LPS-induced TNF-α production in macrophage culture supernatants (Okawara et al., 2014). Diosgenin dose-dependently reduced LPS/IFN-γ-induced NF-κB, JNK, and AP-1 activities to suppress macrophage inflammation, but did not affect TNF-α production (Jung et al., 2010). Also, diosgenin dose-dependently inhibited TNF-α-mediated THP-1 monocyte adhesion and ICAM-1 and VCAM-1 mRNA and protein expression and inhibited VSMC adhesion by inhibiting the MAPK/Akt/NF-κB signaling pathway and ROS production (Choi et al., 2010). Another study reported that diosgenin inhibited thrombosis by downregulating the phosphorylation of NF-κB/p65, IKKβ, Akt, the extracellular signal-regulated kinase (ERK), and JNK, and inhibited TNF-α-induced thrombotic activity and tissue factor (TF) expression in monocytes (Yang et al., 2013).

## Flavonoids

### Soy isoflavones

Soy is the main source of phytoestrogen and has long been used as traditional food. A major phytoestrogen subtype includes isoflavones which have been scientifically validated as benefitting several hormone-dependent conditions (Lephart et al., 2002). Register et al. (2005) in a well-established nonhuman primate AS model, showed how specific soy isoflavone doses generated anti-inflammatory effects specific to soluble vascular cell adhesion molecule-1 (sVCAM-1), whereas the effects of conjugated equine estrogens extended to both sVCAM-1 and MCP-1. It was possible that the athero-protective effects of isoflavones and conjugated equine estrogens were mediated, at least in part by the effects on VCAM-1. A previous study reported that soy isoflavones regulated blood lipids in rats with metabolic syndrome and produced anti-atherosclerosis (Davis et al., 2007).

### Total flavonoids from *Dracocephalum moldavica*

*D. moldavica Linn* (Labiatae) is used in Uyghur medicine and folk to treat coronary heart diseases and hypertension, including AS. Xing et al. (2013) reported that total flavonoids may inhibit TNF-α-induced proliferation and adhesion molecule expression in VSMCs by inhibiting proliferating cell nuclear antigen (PCNA) expression and NF-κB activation in a dose-dependent manner. Thus, *D. moldavica* total flavonoids exhibited anti-inflammatory activities, which is why *D. moldavica* is used for the clinical treatment of AS.

### Resveratrol

Resveratrol exists in TCM such as *Polygonum cuspidatum,* Panax notoginseng, mulberry white skin, *Magnolia officinalis, Shegan,* and plants such as grapes, blueberries, mulberries, and peanuts. The compound is considered a highly effective antioxidant and is bioactive (Biasutto and Zoratti, 2014). Vasamsetti et al. (2016) reported that inhibition of inflammation is the main mechanism by which resveratrol delays the occurrence and development of AS, including inhibition of monocyte differentiation and the production of pro-inflammatory cytokines. Resveratrol could also inhibit the proliferation of VSMCs by promoting the release of Ang-II. The abovementioned mechanisms not only appeared in phorbol myristate acetate (PMA)-induced THP-1 cells but were also fully demonstrated in ApoE−/− mice that had taken resveratrol for 6 weeks. It alleviated the formation of AS plaques and prevented the progression of AS. Chang et al. (2015) reported that resveratrol prevented AS caused by HCD by reducing LDL-C levels and inhibiting atherosclerotic inflammation, as reflected in decreasing the expression of macrophage-specific markers F4/80 and cardiovascular inflammatory marker NF-κB.

### Apigenin

Apigenin (API) is a common flavonoid component found in many foods, with high levels found in celery. API is also abundant in fruit, such as verbena, rhizoma, and selaginaceae. Several previous studies reported that API had antioxidant and anti-inflammatory roles. Ren et al. (2018) reported that no matter whether *in vivo or in vitro,* API had a unique effect in reducing the size of atherosclerotic plaques, and its effective mechanism promotes ATP binding cassette transporter A1 (ABCA1)-mediated cholesterol efflux by inhibiting miR-33 in a time- and dose-dependent manner. At the same time, the phosphorylation of TLR-4, myeloid differentiation primary response protein 88 (MyD88), nuclear factor-κB inhibitor-α (p-IκB-α), and the expression levels of dozens of inflammatory factors such as NF-κB p65 can be reduced by API supplementation, which was achieved by inhibiting the TLR-4/NF-κB signaling pathway. Zeng et al. (2015) reported that API attenuates the inflammation of AS by regulating the apoptosis of macrophages, thereby alleviating the formation of AS, and the mechanism may be to downregulate Plasminogen Activator Inhibitor-2 (PAI-2) by inhibiting the phosphorylation of AKT at Ser473. Zhang et al. (2014) explored the effect and mechanism of API on LPS-induced inflammatory response and found that API can regulate NLRP3 inflammasome assembly, reduce mRNA stability, inhibit macrophage ERK1/2 and NF-κB activation, and finally, LPS-induced levels of IL-6, IL-1β, TNF-α, and other pro-inflammatory cytokines were decreased, indicating that API may slow down the inflammatory response by inhibiting the activation of NLRP3 inflammasome and the production of inflammatory factors.

### Luteolin

Luteolin (3′, 4′, 5, 7-tetrahydroxy flavonoid), as a common natural flavonoid compound in safflower, chrysanthemum, pepper, and other plants, has various biological activities such as curing photoaging (Chen et al., 2012; Lim et al., 2013). Luteolin reduced the expression of inflammatory factors such as ICAM-1, VCAM-1, IL-6, and TNF-α in atherosclerotic ApoE−/− mice fed a high-fat diet and reduced atherosclerotic plaques. However, it showed no effects in ameliorating hyperlipidemia in atherosclerotic ApoE−/− mice. The authors suggested that the mechanism may be related to the enhancement of phosphorylation of signal transducer and activator of transcription 3 (STAT3) (Ding et al., 2019). The 2 μM luteolin pretreatment in LPS-stimulated RAW264.7 cells not only effectively inhibited the occurrence of oxidative stress but also inhibited NLRP3, CARD (ASC), caspase-1, IL-18, and IL-1β inflammatory markers (Zhang et al., 2018). Li et al. (2018) reported that luteolin could inhibit the formation of atherosclerotic plaques by inhibiting the macrophage differentiation marker cluster 68 (CD68), macrophage chemoattractant protein 2 (CCL2), and inflammatory factors. Luteolin also had a therapeutic effect on hyperlipidemia. The mechanism is inseparable from the activation of AMPK-SIRT1 signaling. Jia et al. (2015) emphasized that luteolin inhibited inflammatory markers such as soluble ICAM-1 (sICAM-1) and C-X-C Motif Chemokine Ligand 1 (CXCL1) through the phospho-inhibitor of kappa Bα(IκBα)/NF-κB pathway, thus fully demonstrating the sniping effect of luteolin on vascular inflammation.

### Baicalin

*Scutellaria baicalensis* is a commonly used Chinese herbal medicine used to treat diarrhea, dysentery, hypertension, hemorrhage, insomnia, inflammation, and respiratory tract infections. The main components of *S. baicalensis* are flavonoids, with baicalin as one of the more important active components. Baicalin displayed anti-tumor, antibacterial, and antioxidant effects and improved AS by reducing IL-1, IL-18, mitochondrial ROS, total ROS, and ICAM-1 and VCAM-1 production by inhibiting the NLRP3 inflammasome (Zhao et al., 2020b). Baicalin also inhibited NF-κB and p38 MAPK signaling pathways in a dose-dependent manner, thereby reducing AS-induced increases in IL-6, TNF-α, and soluble vascular endothelial-cadherin (sVE-cadherin) levels, increasing lipolysis-related protein (peroxisome proliferator-activated receptor-α and CPT-1) expression, reducing adipogenesis-related protein (SREBP-1c and ACS) expression, upregulating SOD, catalase (CAT), glutathione peroxidase (GSH-Px) activity, and downregulating MDA activity (Wu et al., 2018a). Baicalin prevented AS by inhibiting the proliferation and migration of ox-LDL-VSMCs by targeting high mobility group box-1 and upregulating miR-126-5p (Chen et al., 2019). Baicalin also inhibited ox-LDL-induced intracellular lipid accumulation and foam cell formation in THP-1 macrophages *via* the peroxisome proliferator-activated receptor gamma (PPARγ)/liver X receptor-α (LXRα)/ABCA1/ATP-binding cassette protein G1 (ABCG1) pathway (He et al., 2016).

### Kaempferol

Kaempferol is a natural edible flavonoid, which is mainly derived from the rhizome of the ginger plant kaempferol, and widely exists in various vegetables and fruits. TNF-α, IL-1β, and MDA levels in atherosclerotic rabbits treated with kaempferol were significantly decreased, serum SOD activity was increased, and the gene and protein expression levels of E-sel, ICAM-1, VCAM-1, and MCP-1 in aortas significantly decreased (Kong et al., 2013). Kaempferol attenuated ox-LDL-induced endothelial cell apoptosis by inhibiting the PI3K/Akt/mTOR pathway and upregulating autophagy (Che et al., 2017). Kaempferol inhibited the TLR4/NF-κB pathway by upregulating the expression of miR-26a, promoted the proliferation of human aortic endothelial cells, and reversed endothelial cell apoptosis caused by ox-LDL (Zhong et al., 2018). Treatment with kaempferol reduced atherosclerotic lesion areas, improved endothelium-dependent vasodilation, and increased maximal diastolic values, while reducing half-maximal effective concentrations, plasma osteopontin (OPN) levels, and aortic OPN and CD44 expression in ApoE^−/−^ mice. Additionally, kaempferol significantly reduced ROS production in the aortas of mice (Xiao et al., 2011). Kaempferol also inhibited macrophage foam cell formation by mediating c-Jun/activator protein-1 (AP-1)-dependent downregulation of CD36 and HO-1-dependent upregulation of SR-BI, ABCA1, and ABCG1 (Li et al., 2013).

## Terpenes

### Taraxacum officinale

*Taraxacum officinale* (TO), as a medicinal and edible homologous plant, dandelion has been used in traditional edible and medicinal applications in my country for thousands of years. It has multiple pharmacological effects such as antioxidant, immune regulation, hypoglycemic, and tumor inhibition (Schutz et al., 2006). The anti-inflammatory and other effects of TO were revealed by its water extract *Taraxacum officinale F. Weber ex Wiggers* (TEE) inhibiting NO production and COX-2 expression *in vivo* and *in vitro* (Jeon et al., 2008). Kim et al. (2000) concluded that TO leaf extract has a good effect on LPS-induced central nervous system inflammation. Jeon et al. (2017) showed that TO extracts could alleviate LPS-induced inflammatory changes in human umbilical vein endothelial cells by inhibiting the NF-κB pathway, which was manifested by reducing the expression of VCAM-1, MCP-1, and pro-inflammatory cytokines.

### Swertiamarin

*Swertiamarin* is a unique kind of *secoiridoid* glycoside isolated from the main bioactive components of the *swedia* family. *Swertiamarin* is the active ingredient of a variety of TCMs, and foreign studies have shown that *Swertiamarin* and its derivatives have anti-diabetic and anti-hyperlipidemic effects (Muhamad Fadzil et al., 2021). Vaidya et al. (2012) reported that *Swertiamarin* reduced serum glucose, triglyceride, non-esterified free fatty acid, and cholesterol levels and also reduced serum MMP-9 and MMP-3 levels when compared with untreated rats. *Swertiamarin* seems to be an excavation point worthy of further study for AS caused by diabetes. Vaidya et al. (2009) also reported that the levels of serum glucose, triglycerides, non-esterified free fatty acids, and cholesterol were effectively reduced in the atherosclerotic rats treated with *Swertiamarin*, and these effects may be attributed to the inhibition of serum MMP-9 and MMP-3 by *Swertiamarin*.

### Geniposide

Gardeniside is a major iridoid glycoside isolated from the dried and ripe fruit of *Gardenia jasminoides*, a Rubiaceae plant. *Gardenia* as TCM has medicinal effects of clearing heat, purging fire, cooling blood, anti-inflammatory and analgesic, and protecting the liver and gallbladder. Wang et al. (2016b) reported that baicalin or geniposide monotherapy and combination therapy, inhibited atherosclerotic lesion development in ApoE^−/−^ mice, reduced the level of inflammatory cytokine IL-12, increased WNT Family Member 1 (Wnt1), and decreased dickkopf-related protein-1 (Dkk1) expression; however, only baicalin or geniposide monotherapy could inhibit the expression of NF-κB, which remained to be investigated, but did not prevent the development of geniposide as an inflammatory target in AS.

### Andrographolide

Andrographolide is the major bioactive component of *Andrographis paniculata* and has various biological properties, including anti-inflammation, anti-oxidation, and anti-hepatotoxicity. Lu et al. (2014) reported that andrographolide could inhibit the proliferation of VECs, relieve inflammation, activate NADPH oxidase, induce heme oxygenase-1 (HO-1) and glutamate cysteine linkage enzyme (glutamate-cysteine ligase, GCLM) expression, and reduce TNFα-induced ICAM-1 expression by inhibiting PI3K/Akt pathway and inhibit VEC proliferation. Chao et al. (2011) held a similar view and they also found that andrographolide reduced inflammation and endothelial cell dysfunction is inseparable from the inhibition of the IKK/NF-κB signaling pathway. Wu et al. (2018b) shared the same view that andrographolide downregulated the expression of MCP-1 and IL-6 by blocking NF-κB signaling in macrophages. They also complimented that the mechanism by which andrographolide reduces AS is also related to the inhibition of foam cell formation and reduction of oxidative stress. Wu et al. (2018c) reported that considering the low oral bioavailability of andrographolide, a well-targeted andrographolide-loaded micelle was prepared using polyethylene glycol and polypropylene sulfide block copolymer as carriers, which effectively inhibited IL-6 and MCP-1 expression, while reduced oxidative stress in macrophages. Al Batran et al. (2014) reported that andrographolide significantly downregulated IL-1β levels in atherosclerotic rabbit serum. The effect of andrographolide in alleviating atherosclerotic inflammation is not only reflected in the inhibition of NF-κB activation but also closely related to the inhibition of STAT3 activation (Lee et al., 2011).

### Artemisinin

*Artemisia annua L*. is an annual medicinal herb and has been used in TCM for a long time. The plant was first thought to have an antipyretic effect by Ge Hong, a medical scientist in the Eastern Jin Dynasty in China. Artemisinin (ART) was successfully extracted from *Artemisia annua L*. and a new antimalarial drug was generated in the 1970s, in China. Cao et al. (2015) showed that ART could treat AS by inhibiting the proliferation, migration, and inflammation of VSMC, which is shown as the inhibition of PCNA, MMP-2, MMP-9, NO, and prostaglandin E2 (PGE2) by ART. Expectations for in-depth research on ART *via* the ROS-NF-kB pathway have been raised, too. Jiang et al. (2016) showed that artesunate (a water-soluble hemisuccinate derivative of ART) had an inhibitory effect on the formation of atherosclerotic plaques and a specific inhibitory effect on LPS-induced IL-8 and MCP-1.

## Quinones

### Tanshinone IIA

*Salvia miltiorrhiza Bunge* is a Lamiaceae plant with dry roots and rhizomes. It was first contained in “*Shen Nong*’*s Materia Medica*.” *Salvia miltiorrhiza* and its components can expand coronary arteries, prevent myocardial ischemia, improve microcirculation, and reduce myocardial oxygen consumption and are widely used in the clinical treatment of cardiovascular diseases. Tanshinone IIA, as the most active lipid-soluble component in *Salvia miltiorrhiza*, has been widely studied for its various biological activities (Jiang et al., 2019). Tanshinone IIA exhibited certain anti-atherosclerotic effects, protected cells from H_2_O_2_ injury (Liu et al., 2014a), suppresses cholesterol accumulation, and affected foam cell formation (Zhang et al., 2011). Stumpf et al. (2013) reported that in the experiment of HUVECs culture, it is found that *Salvia miltiorrhiza* has a good relieving effect on atherosclerotic inflammation and its inhibition of various adhesion molecules, chemokines, and inflammatory mediators such as platelet P-selectin is sufficient evidence. Li et al. (2015) reported that through the cell co-culture model, it was found that Tanshinone IIA had an obvious curative effect on AS and related inflammatory response and can reduce AS-related inflammatory cytokines, which is closely related to the NF-κB signaling pathway.

## Polyphenols

### Curcumin

Curcumin belongs to the class of diketone compounds, mainly derived from the rhizomes of turmeric plants, in the form of orange-yellow crystalline powder, which can be used as food coloring and seasoning. In China, the rhizome of turmeric has a long history of being used as medicine, and it has good menstrual and pain-relieving effects. Modern research has found that curcumin has pharmacological effects such as anti-cancer, anti-inflammatory, anti-oxidation, prevention of senile dementia, and inhibition of AS, with a low price and low toxicity (Araújo and Leon, 2001; Anand et al., 2007; Goel et al., 2008; Gupta et al., 2013). Plasma and liver cholesterol levels and HMG-CoA (3-hydroxy-3-methylglutaryl-CoA) reductase were inhibited in curcumin-fed LDLR−/− mice for 18 weeks. The level of curcumin prevention and treatment of AS is comparable to that of lovastatin (Shin et al., 2011). Curcumin inhibited the expression of MCP-1, increased the expression of ABCA1 and ASR-BI, and increased the excretion of cholesterol, which all showed its preventive effect on AS and its inflammation. The mechanism may be related to the JNK pathway and NF-κB pathway (Liu et al., 2014b). Hasan et al. (2014) reported that curcumin showed a dose-dependent decrease in mouse body weight, plaque formation, and the expression of IL-6 and other inflammatory factors in a low-to-medium dose range. These modulating effects on AS may be related to the inhibition of adipocyte fatty acid binding protein (aP2) and CD36 expression in macrophages by curcumin.

### Magnolol

Magnolol (MAG) is a biphenol compound and the main active ingredient in the TCM *Magnolia officinalis*. MAG reduced VCAM-1 expression on the cell surface and in the cytoplasm (Chen et al., 2002). In 2017, Lee et al. (2017) synthesized a new MAG nanoparticle formulation, which confirmed MAG inhibitory effects on VCAM-1. MAG could prevent early inflammatory lesions of AS by regulating JNK/p38 and NF-κB signaling pathways (Liang et al., 2014).

### Honokiol

Honokiol and MAG are isomers of hydrophobic allyl biphenyl-like structures. Honokiol downregulated TNF-α, IL-6, and IL-1β expression. Additionally, honokiol treatment reduced ROS levels and enhanced SOD activity and also significantly inhibited NO levels, iNOS expression, and the abnormal activation of the NF-κB pathway (Liu et al., 2020). Honokiol significantly inhibited pentraxin 3 (PTX3) overexpression in palm oil-induced HUVECs by inhibiting IKK/IκB/NF-κB signaling pathway. Honokiol also exerted anti-inflammatory effects by significantly inhibiting IL-6, IL-8, and MCP-1 production. It could remind us that PTX3 combined with honokiol may be the crucial inflammation target in treating AS (Qiu et al., 2015). Animal experiments showed that honokiol could inhibit the formation of neointima, the proliferation of smooth muscle cells, and the deposition of extracellular matrix, and its mechanism is closely related to the blocking of NF-κB activation by regulating the ERK signaling pathway (Zhu et al., 2014).

## Others

### Salvianolic acid B

Salvianolic acid B (Sal B) is a highly active ingredient in the water extract of *Salvia miltiorrhiza*. It has antioxidant, anti-inflammatory, and anti-fibrotic effects and has been studied as a potential drug for cardiovascular treatment for many years (Ho and Hong, 2011). Sal B targeted IFN-γ-induced signaling and STAT1 signaling downstream targets to inhibit the expression of CXC chemokines, IFN-γ-inducible protein 10, MIG, and I-TAC and inhibits IP-10 promoter activity and IP-10 protein secretion (Chen et al., 2011). *In vitro* studies using TNF-α to induce monocyte adhesion to HUVECs were performed using different genistein concentrations, with MCP-1 and IL-8 levels tested. The results showed that genistein (0.1 μM) significantly inhibited MCP-1 and IL-8 expression when compared with control cells (Lin et al., 2007).

### Cinnamomum cassia Presl

*Cinnamomum cassia Presl* aqueous extracts were used to study their effects on monocyte differentiation into macrophages and macrophage scavenger receptor activity. In this stage, the aqueous extract of cinnamon can downregulate the expression of scavenger receptor class A (SR-A) and CD36 genes in macrophages, and the inhibition of CD36 expression was concentration-dependent. It was noted that 100 μg/ml cinnamon water extract almost completely blocked macrophage-colony stimulating factor (M-CSF)-induced increases in SR-A protein synthesis (Kang et al., 2014).

### Polysaccharide krestin

*Polysaccharide Krestin* (PSK) is the main active ingredient in *Yunzhi* extract, with molecular weights in the 1.3 × 10^6^ range. As a glucan, the PSK contains β-glycosidic bonds and is detected as β (1→3) and β (1→6) glycosides. Polysaccharides extracted from the mycelium and fermentation broth of Yunzhi polysaccharides strongly inhibit cancer cell activity (Ng, 1998). PSK prevented oxidative damage to macrophages by Ox-LDL and the subsequent foamy degeneration of macrophages (Yuan et al., 1996). In view of the oxidative injury caused by Ox-LDL plays an important role in the transformation of macrophages into foam cells and atherogenesis. PSK protected mouse peritoneal macrophages from oxidative injury by upregulating M-CSF gene expression (Pang, 2003). It represented the front-end preventive effect of PSK on AS from an inflammation perspective.

### Celastrol

*Celastrol* is an effective chemical component extracted from TCM *Tripterygium wilfordii* and other plants and has significant biological activities such as anti-inflammatory and antioxidant (Chen et al., 2018). Allen *et al.* reported that after *Celastrol* loaded into poly (ethylene glycol)-b-poly (propylene sulfide) (PEG-b-PPS) micelles, not only has the scope of treatment been expanded but the number of inflammatory cells and plaque area has been greatly reduced and drug safety has also been better guaranteed (Allen et al., 2019). *Celastrol* reduced the size of atherosclerotic plaques by inhibiting LOX-1 expression and oxidative stress and inhibited various inflammatory factors (Gu et al., 2013). Approximately 100 ng/ml *Celastrol* reduced inflammation and plaque size by regulating Drp1-mediated mitochondrial fission and fusion, ERK1/2, p38, and NF-κB signaling pathways (Tao et al., 2022).

### Tetrahydroxystilbene glucoside

2,3,4′,5-Tetrahydroxystilbene-2-O-beta-d-glucoside (TSG), an analog of resveratrol, is the main active ingredient in the TCM *Polygonum multiflorum* (PMRP) and rhubarb. In recent decades, extensive research has shown that TSG has various pharmacological activities such as antioxidant, free radical scavenging, anti-aging, anti-tumor, neuroprotection, etc. It has also been shown that it also plays an important protective role in cardiovascular diseases (Wu et al., 2017). Li *et al.* showed that PMRP and TSG improved lipid accumulation by reducing the levels of inflammatory factors, glycerol trioleate, and ox-LDL in Apo E−/− mice (Li et al., 2020). TSG could improve the atherosclerotic plaque size and blood lipid level in rats by inhibiting the levels of CRP, IL-6, and other inflammatory factors and the overexpression of MMP-2 and MMP-9 (Zhang et al., 2008b). The high-dose group (120 μg/L) of TSG had the best performance in inhibiting cell adhesion molecules *in vivo* and also had the same performance *in vitro* and reduced the plaque area. It is not negligible that 120 μg/L TSG inhibited ICAM-1/VCAM-1 more strongly than 100 μg/L simvastatin (Wang et al., 2013). Finally, TSG pretreatment at 120 μM inhibited the formation of macrophage foam cells in AS, and the mechanism is related to the inhibition of vimentin expression (Yao et al., 2016).

### Poria polysaccharide

*Poria cocos* is an important homologous raw material used in medicines and foods. *Poria polysaccharide* is the main component in *Poria sclerotia* and has shown great developmental potential in the food, medicine, and healthcare product sectors. PCP inhibited elevated IL-6, TNF-α, NO, LDL, triglycerides, and total cholesterol and also the TLR4/NF-κB pathway activation. PCP also blocked MMP-2 and intercellular adhesion molecule-1 protein expression and interfered with AS in mice (Li et al., 2021). *Poria polysaccharides* interfered with AS by activating the ERK/Nrf2/HO-1 signaling pathway to exert anti-oxidative stress effects (Zhao et al., 2020c).

## Discussion

**FIGURE 2:**
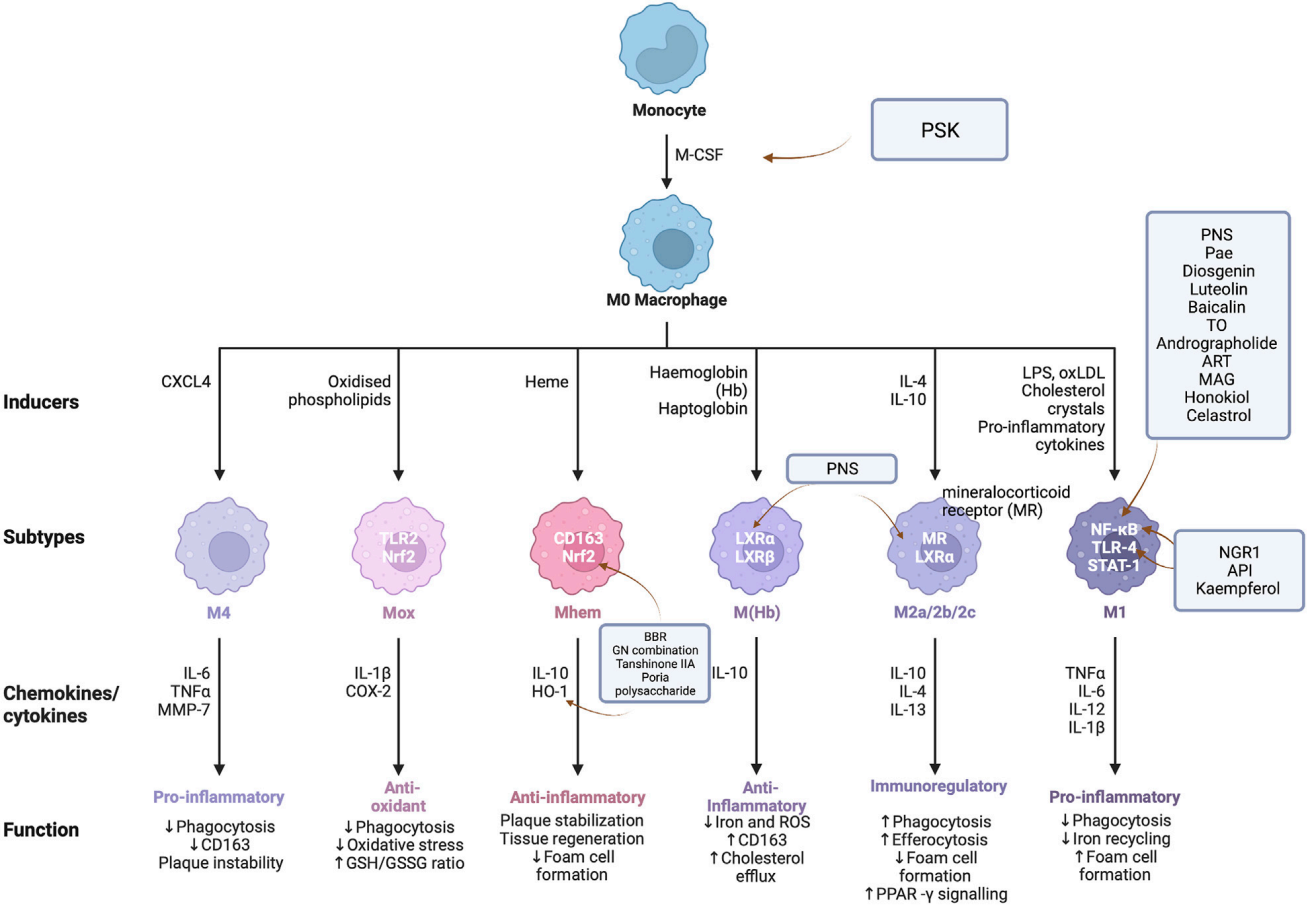
Targets/pathway of natural drugs in as inflammation from the perspective of inflammation, the preventive effect of PSK on AS is to upregulate the gene expression of M-CSF. PNS, Pae, diosgenin, luteolin, baicalin, TO, andrographolide, ART, MAG, Honoriol, Celtrol, and other drugs all participate in the inhibition of NF- κB signal pathway for regulating inflammation of atherosclerosis, while NGR1, API, and kaempferol could inhibit the TLR4/NF- κB signal pathway. BBR, GN combination, tanshinone IIA, and Poria cocos polysaccharide could reduce inflammatory damage by activating Nrf2/HO-1 signal pathway.

Taken together, natural medicines may exert significant anti-atherosclerotic effects by inhibiting inflammation induced by risk factors for AS. Hence, we reviewed the advances in studies from the past ten years on the use of the active components of Chinese herbal medicines to modulate inflammation. We found that 1) current studies show the active ingredients for atherosclerotic mainly in saponins, flavonoids, alkaloids, and terpene; 2) the anti-inflammatory activities of natural drugs mainly focus on TLR4, NF-κB, Nrf2 pathways, and inflammatory factors, such as IL-6, TNF-α, VCAM-1, and MCP-1, as shown in Table 1 and Figure 2; and 3) most previous studies only focused on small molecules derived from plants or herbs for treating AS. Consequently, additional studies could be devoted to investigating potential anti-atherosclerotic macromolecules.

**TABLE 1:** Anti-inflammation effects of natural drugs and the underlying molecular mechanism.

Constituent	Chemical formula	Molecular formula	Study type	Subjects	Mode of administration	Dose	Potential mechanism	References
TMP	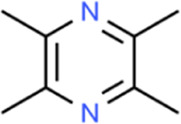	C_8_H_12_N_2_	*In vivo*	In ApoE^−/−^ mice	Intragastric administration	45.05 mg/kg	Inhibiting SCAP/sterol regulatory element-binding protein-1c signaling pathway	Zhang et al. (2017)
			*In vivo*	In Tek-Cre FPN1 mice	Intraperitoneal injection	40 mg/kg	Inhibiting hepcidin, NO, ET-1, ROS, MDA, SOD, IL-1, IL-6, and TNF-α	Sun et al. (2018)
			*In vitro*	In HUVECs	Not mentioned	40 μg/ml	Increasing the production of NO and downregulating the expression of ICAM-1 and HSP60	Wu et al. (2012)
BBR	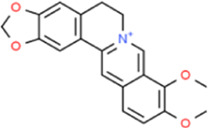	C_20_H_18_NO_4_	*In vivo*	In ApoE^−/−^ mice	Intragastric administration	5 mg/kg	Inhibiting p38 MAPK and JNK signaling pathways	Wan et al. (2018)
			*In vitro*	In HUVECs	Not mentioned	50 μmol/L		
			*In vivo*	In ApoE^−/−^ mice	Oral gavage	78 and 156 mg/kg	Decreasing ROS generation and the serum levels of MDA, ox-LDL, and IL-6	Tan et al. (2020)
			*In vivo*	In ApoE^−/−^ mice	Intraperitoneal injection	10 mg/kg	Suppressing AP-1 activity and activation of the Nrf2/HO-1 pathway	Yang et al. (2020)
			*In vitro*	In THP-1 cells	Not mentioned	1–10 mM		
			*In vitro*	In THP-1 and Raw264.7 cells	Not mentioned	0–50 μM	Increasing miR150-5p level	Lu et al. (2021)
			*In vitro*	In THP-1 cells	Not mentioned	0–40 μg/ml	Regulating the PI3K/AKT/mTOR signaling pathway	Kou et al. (2017)
			*In vitro*	In THP-1 cells and HUVECs	Not mentioned	5–50 μM	Inhibiting expression of VCAM-1 and ICAM‐1	Huang et al. (2013)
			*In vivo*	In ApoE^−/−^ mice	BBR-entrapped nanosystem orally	BBR-entrapped nanosystem orally (100 mg/kg/day)	Decreasing plasma level of TNF-α, IL-6, IL-1β, IFN-γ, MCP, and MIP	Ma et al. (2020)
PL	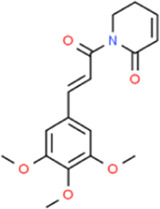	C_17_H_19_NO_5_	*In vitro*	In VSMCs	Not mentioned	1–5 μM	Suppressing PDGF receptor signaling	Son et al. (2012)
PNS	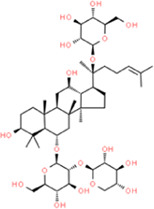	C_47_H_80_O_18_	*In vivo*	In ApoE^−/−^ mice	Intragastric administration	60 mg/kg and 180 mg/kg	Inhibiting the expression levels of NF-κB p65, IL-6, IL-1β, TNF-α, and calpain1 proteins	Yang et al. (2022)
			*In vivo*	In rats	Intraperitoneal injection	100 mg/kg	Enhancing transcriptional activation of the LXRα gene promoter	Fan et al. (2012)
			*In vitro*	In THP-1 cells	Not mentioned	20–80 mg/L, 10–100 mg/L, and 10–100 mg/L		
			*In vivo*	In rats	Intraperitoneal injection	100 mg/kg	Suppressing FAK phosphorylation, integrins expression, and NF-κB translocation	Yuan et al. (2011)
			*In vivo*	In rats	Intraperitoneal injection	100 mg/kg	Decreasing IL-18, IL-1β, and MMP-2 and MMP-9	Zhang et al. (2008a)
			*In vivo*	In ApoE^−/−^ mice	Oral gavage	60 mg/kg	Suppressing the RAGE/MAPK signaling pathway	Dou et al. (2012)
			*In vivo*	In ApoE^−/−^ mice	Aqueous solution of PNS	4 and 12 mg	Decreasing IL-6, TNF-α, ICAM-1, and VCAM-1	Wan et al. (2009)
			*In vitro*	In HCAECs	Not mentioned	30/100/300 μg/ml		
GN combination	Not mentioned	C_17_H_24_O_10_	*In vivo*	In ApoE^−/−^ mice	Intraperitoneal injection	50 + 50 mg/kg	Activating the AMPK/mTOR/Nrf2 signaling pathway	Liu et al. (2021)
		C_47_H_80_O_17_	*In vitro*	In HUVECs	Not mentioned	100μM and 100 μM		
NGR1	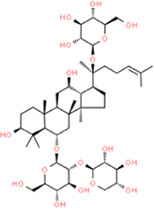	C_47_H_80_O_18_	*In vivo*	In ApoE^−/−^ mice	Intraperitoneal injection	25 mg/kg	Decreasing the levels of IL-2, IL-6, TNF-α, and IFN-γ	Jia et al. (2014)
			*In vivo*	In HUVECs	Not mentioned	30 μM	Regulating XIST/miR-221-3p/TRAF6 axis	Zhao et al. (2020a)
			*In vitro*	In HUVECs	Not mentioned	30 μM	Upregulating miR-221-3p.	Zhu et al. (2020)
			*In vitro*	In HUVECs	Not mentioned	30 μmol/L	Downregulating miR-132	Fu et al. (2018)
ICA	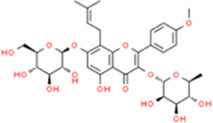	C_33_H_40_O_15_	*In vivo*	In rats	Intragastric administration	30 mg/kg	Inhibiting p38 MAPK signaling Pathway	Hu et al. (2016)
						60 mg/kg		
			*In vitro*	In THP-1 cells	Not mentioned	4, 20 μM	Downregulating CD36 expression and upregulating SR-BI expression	Yang et al. (2015)
			*In vivo*	In ApoE^−/−^ mice	Oral	60 mg/kg	Reducing CX3CR1 and CX3CL1 protein levels	Wang et al. (2016a)
			*In vitro*	In HUVECs	Not mentioned	10, 20, and 40 μmol/L	Reducing the levels of ICAM-1, VCAM-1, and E-sel	Hu et al. (2015)
Pae	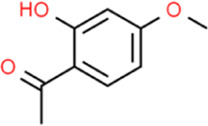	C_9_H_10_O_3_	*In vitro*	In VSMCs and VECs	Not mentioned	120 μM	Inhibiting VEGF and PDGF-B secretion in VECs and Ras-Raf-ERK1/2 signaling pathway in VSMCs	Chen et al. (2014)
			*In vitro*	In VECs	Not mentioned	30, 60, 120, 240, and 480 μM	Promoting miR-126 expression and blocking PI3K/Akt/NF-κB signaling pathway	Yuan et al. (2016)
Diosgenin	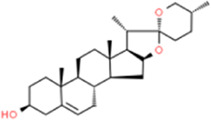	C_27_H_42_O_3_	*In vivo*	In rats	Oral gavage	20 and 40 mg/kg	Regulating AMPK.	Chen et al. (2016)
			*In vitro*	In HUVECs	Not mentioned	0.1, 1, and 10 μmol/L	Blocking IKKβ pathway	Liu et al. (2012)
			*In vivo*	In rats	Intravenous and oral administration	5 mg/ml	Inhibiting LPS-induced TNF-α production in macrophage culture supernatants	Okawara et al. (2014)
			*In vitro*	In Raw264.7 cells	Not mentioned	0.1, 1, and 10 μM	Suppressing the activation of CK2, JNK, NF-κB, and AP-1	Jung et al. (2010)
			*In vitro*	In VSMCs	Not mentioned	0.1–10 μM	Downregulating MAPK, Akt, and NF-κB signaling pathways	Choi et al. (2010)
			*In vitro*	In THP-1 cells	Not mentioned	0.01, 0.1, and 1 μmol/L	Downregulating the phosphorylation of NF-κB/p65, IKK-β, Akt, ERK, and JNK	Yang et al. (2013)
Soy isoflavones	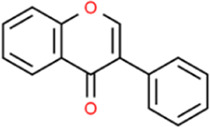	C_15_H_10_O_2_	*In vivo*	In monkeys	Oral	129 mg/d	Inhibiting sVCAM-1	Register et al. (2005)
			*In vivo*	In rats	Not mentioned	230 g/kg	Improving metabolic syndrome in rats	Davis et al. (2007)
Total flavonoids of Dracocephalum moldavica	Not mentioned	Not mentioned	*In vitro*	In VSMCs	Not mentioned	25, 50, and 100 μg/ml	Inhibiting the proliferation, migration, and expression of ICAM-1 and VCAM-1 of VSMCs	Xing et al. (2013)
Resveratrol	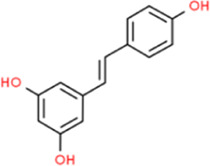	C_14_H_12_O_3_	*In vitro*	In THP-1 cells	Not mentioned	0–100 μmol/L	Decreasing the mRNA levels of IL-1β, TNF-α, and MMP-9	Vasamsetti et al. (2016)
			*In vivo*	In ApoE^−/−^ mice	Oral gavage	10 mg/kg		
			*In vivo*	In ApoE^−/−^ mice	Oral	5 and 25 mg/kg	Reducing NF-κB and macrophage-specific marker F4/80	Chang et al. (2015)
API	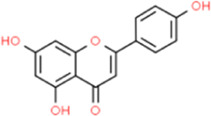	C_15_H_10_O_5_	*In vitro*	In RAW264.7 cells	Not mentioned	0, 10, 20, and 40 μM	Upregulating ABCA1-mediated cholesterol efflux and reducing miR-33, TLR-4, and NF-κB p65 levels	Ren et al. (2018)
			*In vivo*	In ApoE^−/−^ mice	Intragastric administration	2.5 mg/kg		
			*In vitro*	In resident peritoneal macrophages of C57BL/6 mice	Not mentioned	50 μM	Inhibiting AKT Ser473 phosphorylation and downregulating	Zeng et al. (2015)
			*In vivo*	In ApoE^−/−^ mice	Intragastric administration	100 mg/kg	PAI-2	
			*In vitro*	In THP-1 cells	Not mentioned	25 mM	Disrupting NLRP3 and inhibiting ERK1/2 activation	Zhang et al. (2014)
Luteolin	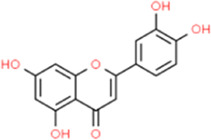	C_15_H_10_O_6_	*In vivo*	In ApoE^−/−^ mice	Oral	10 mg/kg	Inhibiting signal transducer and STAT3	Ding et al. (2019)
			*In vitro*	In primary mouse peritoneal macrophages	Not mentioned	2.5, 5, and 10 μM		
			*In vitro*	In RAW264.7 cells	Not mentioned	0, 1, 2, 4, 8, 16, 32, 64, and 128 μM	Alleviating NLRP3 inflammasome activation and directing macrophage polarization	Zhang et al. (2018)
			*In vivo*	In LDLR^−/−^ mice	Oral	100 mg/kg	Downregulating AMPK‐SIRT1 signaling	Li et al. (2018)
			*In vitro*	In THP-1 cells	Not mentioned	0, 5, 10, and 20 μM		
			*In vivo*	In mice	Oral	Diet containing 0% or 0.6%	Suppressing IΚBα/NF-κB signaling pathway	Jia et al. (2015)
			*In vitro*	In EA. hy926 cells	Not mentioned	0–20 μM		
Baicalin	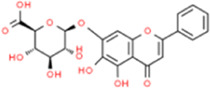	C_21_H_18_O_11_	*In vivo*	In ApoE^−/−^ mice	Intragastric administration	20/50/100 mg/kg	Inhibiting NLRP3 inflammasome	Zhao et al. (2020b)
			*In vivo*	In ApoE^−/−^ mice	Gavage	50 and 100 mg/kg	Inactivating the NF-κB and p38 MAPK signaling pathways	Wu et al. (2018a)
			*In vitro*	In HA-VSMCs	Not mentioned	20 μM	Upregulating miR-126-5p	Chen et al. (2019)
			*In vivo*	In rabbits	Intragastric administration	224 mmol/kg	Regulating the PPARγ-LXRα-ABCA1/ABCG1 pathway	He et al. (2016)
			*In vitro*	In THP-1 cells	Not mentioned	25, 50, and 100 mM		
Kaempferol	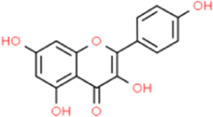	C_15_H_10_O_6_	*In vivo*	In rabbits	Oral	30 and 150 mg/kg	Decreasing E-sel, ICAM-1, VCAM-1, MCP-1, TNF-α, IL-1β, and MDA expressions	Kong et al. (2013)
			*In vitro*	In HUVECs	Not mentioned	50, 100, and 200 μM	Inhibiting the PI3K/Akt/mTOR pathway	Che et al. (2017)
			*In vitro*	In HAECs	Not mentioned	50, 100, and 200 μM	Upregulating miR-26a-5p *via* inhibiting TLR4/NF-κB signaling pathway	Zhong et al. (2018)
			*In vivo*	In ApoE^−/−^ mice	Intragastric administration	50 and 100 mg/kg	Regulating OPN-CD44 pathway	Xiao et al. (2011)
			*In vitro*	In THP-1 cells	Not mentioned	2.5, 5, and 10 μg/ml	Regulating ABCA1, SR-BI, ABCG1, and CD36 expression	Li et al. (2013)
TO	Not mentioned	Not mentioned	*In vitro*	In primary astrocytes	Not mentioned	100 and 1000 mg/ml	Inhibiting TNF-α production from rat astrocytes	Kim et al. (2000)
			*In vitro*	In HUVECs	Not mentioned	100 μg/ml	Inhibiting the NF-κB pathway	Jeon et al. (2017)
Swertiamarin	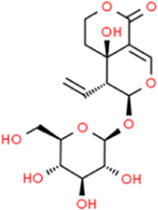	C_16_H_22_O_10_	*In vivo*	In rats	Intraperitoneal injection	75 mg/kg	Decreasing MMP-9 and MMP-3 serum levels	Vaidya et al. (2012)
			*In vivo*	In rats	Oral	50 mg/kg	Decreasing the concentration of TG, TC, and LDL.	Vaidya et al. (2009)
Geniposide	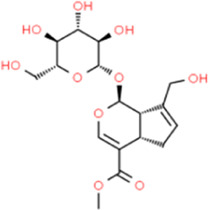	C_17_H_24_O_10_	*In vivo*	In ApoE^−/−^ mice	Gavage	5 mg/ml	Increasing Wnt1 and inhibiting DIKK1 expression	Wang et al. (2016b)
Andrographolide	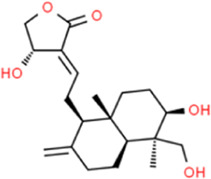	C_20_H_30_O_5_	*In vitro*	In EA. hy926 cells	Not mentioned	7.5 μM	Regulating PI3K/Akt/Nrf2 and PI3K/Akt/AP-1 pathways	Lu et al. (2014)
			*In vitro*	In EA. hy926 cells	Not mentioned	10 μM	Inhibiting ICAM-1 expression and NF-κB activation	Chao et al. (2011)
			*In vitro*	In RAW 264.7 cells	Not mentioned	0, 1.25, 2.5, 5, 10, 20, and 40 μM	Suppressing pro-inflammation and ROS generation-mediated foam cell formation	Wu et al. (2018b)
			*In vitro*	In RAW 264.7 cells	Not mentioned	3.5 μg/ml	Downregulating IL-6 and MCP-1	Wu et al. (2018c)
			*In vivo*	In rabbits	Oral	10/20 mg/kg	Reducing the expression of CD36	Al Batran et al. (2014)
			*In vitro*	In RAW 264.7 cells	Not mentioned	0, 2, 10, and 50 μM	Inhibiting the activation of NF-κB and STAT3	Lee et al. (2011)
ART	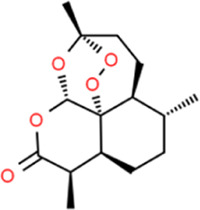	C_15_H_22_O_5_	*In vitro*	In HUVECs	Not mentioned	100 μM	Inhibiting the NF-κB pathway	Cao et al. (2015)
			*In vivo*	In ApoE^−/−^ mice	Intramuscular injection	1.5, 5, and 15 mg/kg/day	Downregulating TNF-α, IL-6, IL-8, and MCP-1 expressions	Jiang et al. (2016)
Tanshinone IIA	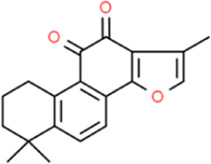	C_19_H_18_O_3_	*In vivo*	In ApoE^−/−^ mice	Intragastric administration	30 mg/kg/day	Regulating ERK/Nrf2/HO-1 pathway	Liu et al. (2014a)
			*In vitro*	In THP-1 cells	Not mentioned	1–10 μM		
			*In vitro*	In RAW 264.7 cells	Not mentioned	30 μg/ml	Regulating the SR-BI and cholesteryl ester-TG interchange with TG-rich lipoproteins pathway	Zhang et al. (2011)
			*In vitro*	In HUVECs	Not mentioned	0, 100, 200, 250, and 400 μg/ml	Reducing TNF-α, VCAM-1, ICAM-1, IL-6, IL-8, and MCP-1	Stumpf et al. (2013)
			*In vitro*	In HUSMCs	Not mentioned	10 μg/ml	Reducing TNF-α, MCP-1, ET-1, NO, ICAM-1, and IL-10	Li et al. (2015)
Curcumin	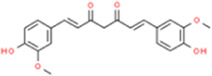	C_21_H_20_O_6_	*In vivo*	In LDLR (−/−) mice	Oral	0.02% w/w curcumin	Upregulating PPARγ and LXRα expression	Shin et al. (2011)
			*In vitro*	In RAW 264.7 cells	Not mentioned	0, 5, 10, 20, and 40 μM	Suppressing the JNK pathway	Liu et al. (2014b)
			*In vivo*	In LDLR (−/−) mice	Oral	500, 1,000, and 1,500 mg/kg	Suppressing aP2 and CD36 expression	Hasan et al. (2014)
MAG	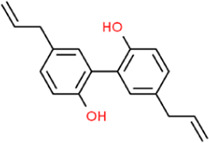	C_18_H_18_O_2_	*In vitro*	In HAECs	Not mentioned	5 μM	Attenuating VCAM-1 expression	Chen et al. (2002)
			*In vivo*	In rabbits	Intramuscular injection	1 μg/kg		
			*In vitro*	In HUVECs	Not mentioned	10 μM	Mediating ERK, AKT, and NF-κB signaling pathways	Lee et al. (2017)
			*In vitro*	In HAECs	Not mentioned	5 μM	Inhibiting JNK/p38 and NF-κB signaling pathways	Liang et al. (2014)
			*In vivo*	In ApoE^−/−^ mice	Intraperitoneal injection	10 mg/kg/day		
Honokiol	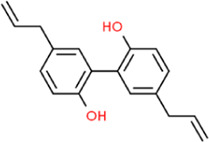	C_18_H_18_O_2_	*In vivo*	In ApoE^−/−^ mice	Injection	10 and 20 mg/kg	Downregulating TNF-α, IL-6, IL-1β, and ROS.	Liu et al. (2020)
			*In vitro*	In HUVECs	Not mentioned	10 μM	Suppressing PTX3 overexpression	Qiu et al. (2015)
			*In vitro*	In RASMCs	Not mentioned	2, 2.5, and 10 μM	Blocking NF-κB activation *via* the ERK signaling pathway	Zhu et al. (2014)
Sal B	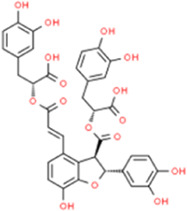	C_36_H_30_O_16_	*In vitro*	Bovine aortic endothelial cells	Not mentioned	0, 1, 10, 25, and 50 μM	Suppressing IFN-γ-induced JAK/STAT1 activation	Chen et al. (2011)
			*In vivo*	In ApoE^−/−^ mice	Oral	0.3% Sal B	Attenuating MMP-2 and MMP-9 expression	Lin et al. (2007)
			*In vitro*	In HASMCs	Not mentioned	10 mM		
Cinnamomum cassia Presl	Not mentioned	Not mentioned	*In vitro*	In THP-1 cells	Not mentioned	10, 50, and 100 μg/ml	Suppressing the upregulation of SR-A by M-CSF and modulated ERK1/2 activity	Kang et al. (2014)
PSK	Not mentioned	Not mentioned	*In vitro*	In mouse peritoneal macrophages	Intraperitoneal injection	3 mg/per mouse	Protecting macrophages from lipoperoxide accumulation and foam cell formation caused by ox-LDL.	Yuan et al. (1996)
			*In vitro*	In mouse peritoneal macrophages	Not mentioned	0.2 ml	Upregulating M-CSF gene expression	Pang, (2003)
Celastrol	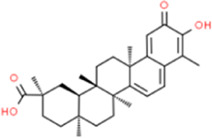	C_29_H_38_O_4_	*In vivo*	In LDLR (−/−) mice	Tail vein injection and intraperitoneal injection	Celastrol micelle formulations (500 μl)	Inhibiting NF-κB signaling	Allen et al. (2019)
			*In vitro*	In RAW 264.7 cells	Not mentioned	10 ng/ml or 1 μg/ml		
			*In vitro*	In RAW 264.7 cells	Not mentioned	50–200 nM	Inhibiting LOX-1 and oxidative stress	Gu et al. (2013)
			*In vitro*	In RAW 264.7 cells	Not mentioned	100 nmol/L	Regulating Drp1-dependent mitochondrial fission and fusion, ERK1/2, p38, and NF-κB signaling pathways	Tao et al. (2022)
TSG	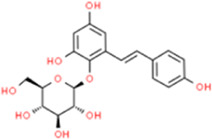	C_20_H_22_O_9_	*In vivo*	In ApoE−/− mice	Not mentioned	0.035 and 0.07 mg/g per day	Downregulating IL-6, TNF-α, VCAM-1, and MCP-1 expression	Li et al. (2020)
			*In vivo*	In rats	Oral gavage	120, 60, or 30 mg/kg per day	Suppressing the expression of MMP-2 and MMP-9	Zhang et al. (2008b)
			*In vivo*	In rats	Oral gavage	120, 60, or 30 mg/kg per day	Suppressing the expression of ICAM-1/VCAM-1	Wang et al. (2013)
			*In vitro*	In U937 cells	Not mentioned	120, 60, and 30 μg/L		
			*In vitro*	In U937 cells	Not mentioned	10, 25, 50, and 100 μM	Suppressing vimentin expression and cleavage, adhesion molecules expression, and vimentin-ICAM-1 co-localization	Yao et al. (2016)
Poria polysaccharide	Not mentioned	Not mentioned	*In vivo*	In ApoE−/− mice	Intragastric administration	100, 200, and 400 mg/kg	Inhibiting TLR4/NF-κB pathway and blocking MMP-2 and ICAM-1	Li et al. (2021)
			*In vitro*	In VSMCs	Not mentioned	50, 100, and 200 μg/ml	Activating Nrf2/HO-1 signaling pathway by ERK.	Zhao et al. (2020c)

Although, many studies have been conducted on the ability of natural medicines to inhibit inflammation and most of them have reached a certain depth. However, some problems persist, and many studies should be performed in the future. First, the current research is mostly superficial, based on the results of *in vitro* or *in vivo* experiments; therefore, further clinical experiments should be provided conclusive evidence for the anti-atherosclerotic effect of natural drugs. Second, natural medicines have many problems with themselves. For example, as an effective pharmacological component of Ligusticum chuanxiong root, the content of TMP is not high in raw medicine, and even sometimes undetectable in Ligusticum chuanxiong root; ICA may also have problems such as chronic toxicity, subchronic toxicity, reproductive toxicity, and developmental toxicity. Third, compared with the extensive pharmacological effect of natural drugs, the practical application of natural drugs as clinical medicine is relatively small, the biggest problem is the low bioavailability. Therefore, we can adopt the following methods in future research; on one hand, we could optimize the matrix materials to increase the drug loading of new dosage forms and improve the stability of the reparations. On the other hand, we need to strengthen the evaluation of pharmacokinetics and pharmacodynamics of new preparations to improve preclinical research of drugs and promote clinical trials of new preparations.

In conclusion, we hope that this review will provide a reference for understanding the current potential of natural drugs and their pharmacological mechanisms and highlight the advantages of these natural drugs in the prevention and treatment of AS by regulating inflammation.
